# Prevalence and Determinants of Multimorbidity, Polypharmacy, and Potentially Inappropriate Medication Use in the Older Outpatients: Findings from EuroAgeism H2020 ESR7 Project in Ethiopia

**DOI:** 10.3390/ph14090844

**Published:** 2021-08-25

**Authors:** Akshaya Srikanth Bhagavathula, Mohammed Assen Seid, Aynishet Adane, Eyob Alemayehu Gebreyohannes, Jovana Brkic, Daniela Fialová

**Affiliations:** 1 Department of Social and Clinical Pharmacy, Faculty of Pharmacy in Hradec Králové, Charles University, 500 05 Hradec Králové, Czech Republic; jovanabrkic37@gmail.com (J.B.); fialovad@faf.cuni.cz (D.F.); 2 Department of Clinical Pharmacy, School of Pharmacy, College of Medicine and Health Sciences, University of Gondar, Gondar P.O. Box 196, Ethiopia; hassenm100@gmail.com; 3 Department of Internal Medicine, College of Medicine and Health Sciences, University of Gondar, Gondar P.O. Box 196, Ethiopia; ayne.2003@yahoo.com; 4 Division of Pharmacy, School of Allied Health, University of Western Australia, Crawley, WA 6009, Australia; justeyob@gmail.com; 5 Department of Geriatrics and Gerontology, 1st Faculty of Medicine, Charles University, 120 00 Prague, Czech Republic

**Keywords:** multimorbidity, polypharmacy, potentially inappropriate medication use, older adults, prevalence, determinants, chronic, outpatient, 2019 Beers criteria, Ethiopia

## Abstract

Few studies have been conducted on multimorbidity (two or more chronic diseases) and rational geriatric prescribing in Africa. This study examined the prevalence and determinants of multimorbidity, polypharmacy (five or more long-term medications), and potentially inappropriate medication (PIM) use according to the 2019 Beers criteria among the older adults attending chronic care clinics from a single institution in Ethiopia. A hospital-based cross-sectional study was conducted among 320 randomly selected older adults from 12 March 2020 to 30 August 2020. A multivariable logistic regression analysis was performed to identify the predictor variables. The prevalence of multimorbidity, polypharmacy, and PIM exposure was 59.1%, 24.1%, and 47.2%, respectively. Diuretics (10%), insulin sliding scale (8.8%), amitriptyline (7.8%), and aspirin (6.9%) were among the most frequently prescribed PIMs. Older patients experiencing pain flare-ups were more likely to have multimorbidity (adjusted odds ratio (AOR): 1.64, 95% confidence intervals: 1.13–2.39). Persistent anger (AOR: 3.33; 1.71–6.47) and use of mobility aids (AOR: 2.41, 1.35–4.28) were associated with polypharmacy. Moreover, cognitive impairment (AOR: 1.65, 1.15–2.34) and health deterioration (AOR: 1.61, 1.11–2.32) increased the likelihood of PIM exposure. High prevalence of multimorbidity and PIM use was observed in Ethiopia. Several important determinants that can be modified by applying PIM criteria in routine practice were also identified.

## 1. Introduction

The World Health Organization (WHO) defined multimorbidity as the coexistence of two or more chronic conditions in the same individual [1]. With a growing proportion of the older population, the health burden of multimorbidity is expected to increase more rapidly [2]. In general, multimorbidity among older people often leads to the use of multiple medications (also known as polypharmacy) and increases the risk of potentially inappropriate medication (PIM) use. Although there is no universal definition for polypharmacy, the most commonly used WHO definition is the concurrent use of five or more different medications [3]. In contrast, PIM use is defined as the use of “medication/medication class that should generally be avoided in people aged 65 years or older because they are either ineffective or pose unnecessary high-risk for such age group where a safer alternative is available” [4]. Moreover, multimorbidity simultaneously increases the use of multiple medications, and inappropriate use of multiple medications can lead to adverse drug events and increase morbidity and mortality in older patients [2,4]. Thus, optimizing pharmacotherapy and assessing the appropriateness of prescriptions in the older population have become global public health concerns.

Several studies have shown the effectiveness of comprehensive medication reviews in older people in reducing the number of medication-related problems and PIM use [5,6,7,8,9]. Over the past two decades, several evidence-based screening tools have been developed to avoid PIM use in older patients and prevent medication-related harms [10,11,12]. Mark Beers and associates developed the Beers Criteria in 1991, with several revisions made in 1997, 2003, 2012, 2015, and 2019 [13]. The American Geriatrics Society (AGS) approved and updated the Beers criteria in 2019 with several new modifications, clarifications of criteria, definitions, and explanations to ensure appropriate medication use among older adults and avoid adverse events associated with polypharmacy and PIM use [14]. However, the clinical use of AGS Beers criteria 2019 in improving medication appropriateness in older patients in African countries has not yet been determined.

Ethiopia is a sub-Saharan African (SSA) developing country located at the horn of Africa. The United Nations (UN) estimated that individuals aged 65 years and older accounted for 3.6% of the Ethiopian population in 2020 and is expected to reach 5.2% by 2050 [15]. Previous studies have investigated the extent of polypharmacy and PIM use using START/STOPP criteria and Beers criteria [16,17,18,19,20,21] and found poor medication-related quality of life among Ethiopian older patients [22]. Moreover, a recent meta-analysis from Ethiopia reported a high prevalence of PIM use (37%) among the older population [23]. However, the prevalence and determinants of multimorbidity, polypharmacy, and PIM use in the older population have not been previously evaluated. Thus, the objective of this study was to evaluate the prevalence and determinants of multimorbidity, polypharmacy, and PIM use, using the updated AGS Beers criteria 2019, in older patients attending chronic care outpatient clinics in Ethiopia.

## 2. Results

### 2.1. Demographic Characteristics of Study Participants

In this study, 320 older patients (aged 65 and above) participated with a response rate of 100%. The mean age of the study population was 71.9 (SD: 6.07) years. The majority of the subjects were men (59%), illiterate (65%), and married (70.3%). Table 1 shows the main sociodemographic and clinical characteristics of patients and the comprehensive geriatric assessment (CGA) variables. Among chronic conditions, the majority of patients had hypertension (66.6%), diabetes mellitus (36.8%), and other diseases (22.5%). The average CCI score was 2.53 (SD: 1.38), and the mean (SD) number of medications per patient was 3.4 (SD: 1.69). Around 40% of the participants had a history of hospitalization. Serum creatinine data were available for 188 patients (58.7%), and 13.1% of them had creatinine clearance (CrCl) levels of <30 mL/min.

The CGA results revealed that most participants were able to perform independently activities of daily living (85%) and understood verbal and non-verbal communications (72.8%). However, some had repeated health complaints (21.9%), experienced a fall in the past year (13.8%) and had cognitive deficiencies (17.5%) (Table 1).

### 2.2. Prevalence of Multimorbidity, Polypharmacy, and PIM Use

**Figure 1:**
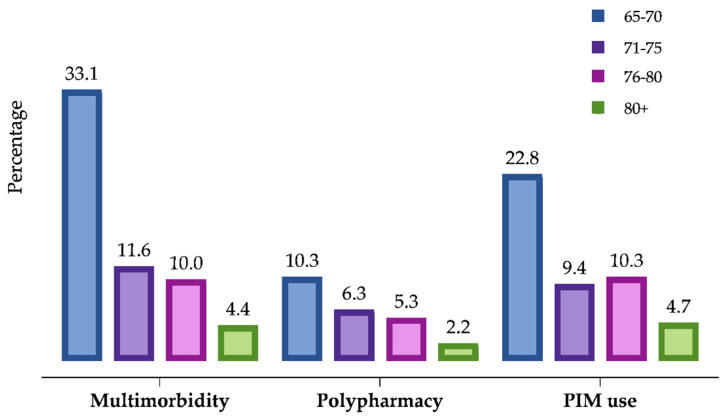
Distribution of multimorbidity, polypharmacy, and PIM use in older population.

The overall prevalence of multimorbidity was 59.1% (95% CI: 53.5–64.5), polypharmacy was 24.1% (95% CI: 19.4–29.3), and PIM exposure based on the AGS Beers criteria 2019 list was 47.2% (95% CI: 41.6–52.8). The majority of the patients, aged 65–70 years, had higher prevalence of multimorbidity (33.1%, 95% CI: 28–38.6), polypharmacy (10.3%, 95% CI: 7.2–14.2), and PIM use (22.8%, 95% CI: 18.3–27.8). The prevalence of multimorbidity, polypharmacy, and PIM use across different age groups is shown in Figure 1.

**Figure 2:**
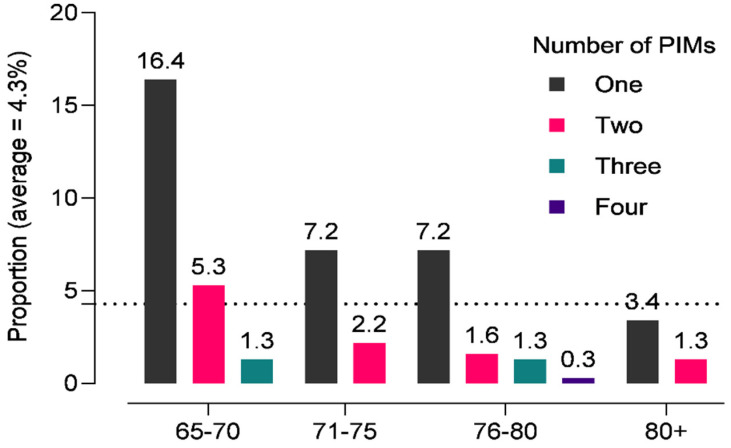
Proportion of older adults receiving potentially inappropriate medications according to 2019 AGS Beers criteria in an outpatient setting in Ethiopia.

A total of 203 PIMs were identified in 151 participants according to the AGS Beers Criteria 2019, and 34.1% of participants were prescribed at least one PIM, while 10.4% were prescribed two PIMs. However, prescribing three (2.6%) or four (0.3%) PIMs was uncommon. Figure 2 illustrates the proportion of individuals per age group who were prescribed a PIM.

The most commonly prescribed PIMs in the older patients were antidiabetic medications (13.5%), cardiovascular medications (9.7%), and antidepressants (7.8%). Potential drug–drug interactions were found in 2.8% of the cases. Moreover, most of the PIMs prescribed (24%) were medication classes to be used with caution among older adults aged ≥70 years (19.7%), particularly diuretics and aspirin for the primary prevention of cardiovascular events. Furthermore, 11.4% of the participants with reduced CrCl of <30 mL/min were exposed to PIMs (Table 2).

### 2.3. Determinants of Multimorbidity, Polypharmacy, and PIM Use

Bivariate and multivariate logistic regression analyses were performed to assess the factors that exhibit significant association with multimorbidity, polypharmacy, and PIM prescription. Although we found several significant factors associated with outcome variables in the crude analysis, these results slightly changed after adjusting for baseline variables (age, gender, education, marital status, and CCI score) (Table 3).

#### 2.3.1. Multimorbidity

Older patients suffering from pain flare-ups were associated with multimorbidity (AOR: 1.64, 95% CI: 1.13–2.39).

#### 2.3.2. Polypharmacy

Older patients who could understand verbal and non-verbal cues (AOR: 2.06, 95% CI: 1.02–4.17), used walking assistance devices (AOR: 2.41, 95% CI: 1.35–4.28), presented higher levels of anger (AOR: 3.33, 95% CI: 1.71–6.47), and experienced flare-up in pain (AOR: 2.00, 95% CI: 1.03–3.89) were more likely to have polypharmacy prescriptions.

#### 2.3.3. PIM Use

PIM use was significantly associated with several factors, such as the use of walking assistance devices (AOR: 1.83, 95% CI: 1.32–2.53), cognitive impairments (AOR: 1.65, 95% CI: 1.15–2.34), poor health (AOR: 1.61, 95% CI: 1.11–2.32), and lack of interest in activities (AOR: 1.46, 95% CI: 1.00–2.14). Interestingly, older patients’ abilities to understand verbal and non-verbal communications were associated with lower odds for PIM exposure (AOR: 0.17, 95% CI: 0.03–0.80). More details are given in Table 3.

## 3. Discussion

This study sought to assess the prevalence and determinants of multimorbidity, polypharmacy, and PIM use among older patients attending chronic care clinics in Ethiopia. Overall, the prevalence of multimorbidity and PIM use is becoming increasingly common in Ethiopia, and our findings are in concordance with other studies [18,19,20,23,24]. It was found that around 60% of the older adults had multimorbidity, a quarter of them experienced polypharmacy, and nearly half (47.1%) of them were being treated with at least one PIM during the study period. This highlights the need for multidisciplinary care in older patients and rational geriatric prescribing practices to minimize drug-related problems (DRPs).

To the best of our knowledge, this is the first study that applied the complete version of the 2019 Beers criteria to an SSA older population. None of the recent studies that have reported PIM use in the older population in West Africa [25], Nigeria [26,27], and South Africa [28] have used the 2019 AGS Beers criteria [10]. Furthermore, recently published studies from Ethiopia have used 2015 and 2019 criteria but have not reported the medications that are needed to avoid or to be used cautiously in the older population [29,30]. However, the current estimates of the prevalence of PIM use using the 2019 AGS Beers criteria were generally higher than those reported by previously published studies in Ethiopia using 2015 STOPP/START criteria and 2012 Beers criteria, reporting inappropriate medication use in 23% to 45% of the participants [20,31]. In addition, international studies using the 2019 AGS Beers criteria reported a high prevalence of PIM use in patients with heart failure in Lebanon (80%) [32], outpatients in Qatar (76%) [33], and patients with diabetes in India (74%) [34].

The most common classes of PIMs in our study were cardiovascular medications (27.8%), followed by endocrine agents (13.5%). In contrast, 84.2% of the PIMs were gastrointestinal medications in a Qatar study [33], and 44.6% were endocrine agents in India [34]. Other studies have reported different classes of PIMs, such as benzodiazepines [35], antidepressants and antipsychotics [36], and non-steroidal anti-inflammatory drugs [37]. The differences in PIM medication classes may be related to different study populations, study settings, availability of medications, and the criteria applied to identify PIMs. Based on our findings and in line with findings of other studies [33,34,35,36,37], it is crucial to reduce the overuse of unnecessary medications in older patients with multiple morbidities. Furthermore, our study revealed a considerable difference in the prevalence of multimorbidity and PIM use in different age groups and lower prevalence of polypharmacy across all age groups. This paradox in our study reflects the important differences between developed and developing countries, as most physicians in developing countries do not apply the principles of geriatric prescribing due to low awareness of explicit criteria.

Consistent with other studies, the multivariate logistic regression identified several age-specific factors associated with multimorbidity, polypharmacy, and PIM use [38,39,40,41,42,43]. As a rule of thumb, many body functions decline in old age, which is a frequent cause of multimorbidity [1,10,14,44,45]. The flare-up of nonspecific pain in older adults appears to be a principal component of multimorbidity and has higher odds of co-occurring with polypharmacy [37], this suggesting that pain flare-up predisposes one to polypharmacy. For example, 17.5% of participants in our study reportedly experienced pain most of the days, associated with higher odds of multimorbidity and polypharmacy. In addition, the study finds that an increase in the number of medications used in older patients was associated with persistent anger. A study documented that the increase in the number of depressive symptoms over time in older patients resulted from polypharmacy, social impairment, and behavioral agitation [46]. The study also found that medication review at baseline showed a moderated positive impact on social functioning and polypharmacy on depression was subsided over time [46]. However, the mechanism showing that polypharmacy causes anger was not clearly explored in the literature. The possible explanation could be that older people on polypharmacy may develop a sense of helplessness about their health status and stimulate more negative feelings, leading to behavioral agitation.

Furthermore, several factors related to geriatric syndrome, such as the use of mobility aids, functional limitations, distress, deterioration of health, and lack of interest in activities of daily living, are significant predictors of polypharmacy and PIM exposure in our study. Understanding oral and written communication was associated with > 50% lower odds of being prescribed PIMs. The reason for this may be that older patients can elicit and understand medication-related information and communicate information to their physicians. Thus, it has an advantage of perceived self-efficacy in obtaining information and attention to their medical concerns from physicians.

Polypharmacy is considered to be an important marker of multimorbidity and PIMs; it is like a double-edged sword. In our study, older patients experiencing flare-ups in pain in older population was associated with multimorbidity and polypharmacy. While it may be difficult to establish a causal relationship, our study’s findings clearly show that polypharmacy is linked with chronic pain and inflammation that can result in morbidity and subsequently reduce the quality of life in older adults. However, developing a validated instrument or checklist to understand the cascade of multimorbidity, polypharmacy, and PIM use can subsequently help optimize medication use and improve rational drug prescribing in the older population.

Although RAS inhibitors and diuretics are deemed safe in the older population, the 2019 AGS Beers criteria recommended to avoid using them in older patients with reduced renal function [14]. In our study, around 16% of the PIMs are due to these medications. A Spanish study found an association of RAS inhibitors—with or without other drugs—with increased nephrotoxicity risk (17%) [47]. 

The present study also showed that 28 patients (8.8%) reported the insulin sliding scale as a PIM according to 2019 AGS explicit criteria. The insulin sliding scale is an agent approved for use in diabetic patients; however, in older patients, it may have a higher risk of hypoglycemia without an improvement in hyperglycemia management, and it is now recommended to avoid using it in older adults as per the 2019 AGS Beers criteria. In addition, a study from Oman using the 2015 Beers criteria identified amitriptyline (11%) among the top PIMs in the older patients [48], and a systematic review of Guaraldo et al. found that six out of seven studies (85.7%) mentioned amitriptyline among the most used PIMs in the older population [49]. The inappropriate medication use in Ethiopia could be due to low awareness of physicians about the risk of PIMs in older patients (disregarding the level of multimorbidity and polypharmacy) and lack of applicability of explicit criteria in their prescribing practice.

### Limitations

This study has some limitations. First, this is a hospital-based cross-sectional study conducted in a single institution that did not show cause–effect relationships and cannot be generalized to other populations in Ethiopia. Second, some independent variables, such as patient characteristics, were self-reported and collected using a standardized tool. However, the accuracy of the information depends on subjects’ abilities to recall events, and bias related to patients’ forgetfulness or possible unwillingness to share information could not be ruled out. Low income and education levels limit the ability of self-report on certain health conditions and effort in clinical investigations, such as serum creatinine tests. Due to some missing data of some of the variables such as serum creatinine (*n* =188) and history of hospitalization, we could include them in the logistic regression. Since the study was cross-sectional, a causal relationship could not be established. Moreover, inpatient facilities may have higher rates of multimorbidity, polypharmacy, and PIM use than outpatient settings [50]. Last, the appropriateness of the medication use was evaluated using the 2019 AGS Beers criteria, and the prevalence of certain PIMs, which were not included in the criteria list previously, might have been overestimated.

## 4. Materials and Methods

A cross-sectional study was conducted among older patients (≥65 years) attending the ambulatory care clinics from 12 March 2020, to 30 August 2020, in the University of Gondar (UOG) Teaching Hospital, Gondar, Northwest Ethiopia. The UOG Teaching Hospital, founded in 1954, is providing services to five million people living in and around Gondar. It is one of the biggest comprehensive specialized hospitals in the Amhara region and a major referral hospital for eight government healthcare centers. The UOG chronic care clinics provide services to patients with diabetes, cardiovascular diseases, hypertension, psychiatric issues, and other chronic diseases. It is estimated that around 10,000 patients with cardiovascular diseases and 8000 patients with diabetes have chronic illnesses follow-up every month.

This research was conducted as part of the EuroAgeism H2020 ESR7 project entitled “Inappropriate prescribing and availability of medication safety and medication management services in older patients in Europe and developing countries”. The EuroAgeism ESR7 project, a multinational cross-sectional study, aimed to evaluate the rationality of drug prescribing in older patients in eight countries: the Czech Republic, Serbia, Estonia, Bulgaria, Croatia, Spain, Turkey, and Ethiopia.

### 4.1. Sample Size and Sampling Technique

The required sample size was calculated via Open Epi software using a single population proportion formula with the following assumptions of 27.7% prevalence of PIM use in older people in Gondar, Ethiopia [16], 95% confidence level, and 5% margin of error. Therefore, the total calculated sample size was 308. Older patients attending chronic care clinics who agreed to participate in a CGA during the study period and provided written informed consent were included in the study.

### 4.2. Data Collection

Research assistants conducted data collection and received intensive training on study tools, data collection methods, and ethical concerns. The data collection tools were pilot tested on 15 randomly selected patients before starting the actual data collection process. The data collection tools were translated to the local language (Amharic) with modifications and back-translated to English to conform to its original meanings. The research process was checked weekly, and data collection was performed under the supervision of the principal investigator and co-investigators. Eligible patients were approached for informed consent by research assistants during patients’ visits to chronic care clinics. All patients who consented to participate completed a ESR7 study protocol (including CGA) administered by the research assistants, unaware of the study’s aim and hypothesis. The data collection included (1) sociodemographic variables (age, gender, education, marital status, and living arrangements); (2) body mass index (BMI), history of medical problems, medications used, and information on recent hospitalizations were recorded and cross-checked with the patient medical records; (3) geriatric health assessment including diseases, symptoms, and other relevant information about health status, medication use, recent results of laboratory tests, adherence to medications, and questions related to the quality of life and satisfaction with provided care were also part of the study protocol. All assessments were completed in a separate room, and the collected data were de-identified by giving a unique code in database and paper forms.

Although a large number of data variables were collected following the EuroAgeism ESR7 study protocol, a set of other CGA variables related to i.e., the understanding of verbal and non-verbal communication, physical fitness, health and functional status, cognitive performance, barriers to physical and social activities, and dealing with pain flare-ups were considered also for this study. Several operational definitions were followed during the data collection and interpretation of the study findings.

- Multimorbidity is defined as the presence of two or more long-term conditions that cannot be cured but can be controlled through medications or other treatments [1].
- Polypharmacy is considered if the patient is taking at least five medications regularly [3].
- PIM use is defined as drug therapy whose potential risks outweigh potential benefits, and identified PIMs were classified according to the 2019 AGS Beers criteria [14].
- Charlson comorbidity index (CCI) score is used to measure the severity of the comorbidity for each patient quantitatively [43]. Patients were divided into three groups: mild, with a CCI score of 1–2; moderate, with a CCI score of 3–4; and severe, with a CCI score of ≥5.

### 4.3. Statistical Analysis

The collected data were visually checked for completeness and were coded and entered into MS Excel for Windows and exported to SPSS for analysis. Descriptive statistics, such as frequency, percentages, means, and standard deviations (SD), were conducted to describe the study population in relation to different variables. The prevalence rates of multimorbidity, polypharmacy, and PIM use in the older population were calculated for each age group. The number of PIMs identified in each age group was documented using the 2019 AGS Beers criteria. Univariate and multivariate regression analyses were conducted separately to identify the determinants (CGA variables) of multimorbidity, polypharmacy, and PIM exposure. Independent variables, such as age, gender, marital status, education, and CCI scores, were included in the adjusted multivariable regression model. An adjusted odds ratio (AOR) with a 95% confidence interval (CI) was used to measure the associations. A two-sided *p*-value of < 0.05 was considered statistically significant.

## 5. Conclusions

The study found that the prevalence of multimorbidity and PIM use among Ethiopian older adults was substantially high in an outpatient setting. The research identified several important determinants that could increase the risk of DRPs in the Ethiopian older population. These findings stress the need for multifaceted, interdisciplinary interventions to multiple chronic disease conditions; awareness of PIMs; and improvement of rational geriatric prescribing in Ethiopia.

## Figures and Tables

**Table 1:** Characteristics of ambulatory older patients with multimorbidity, polypharmacy, and PIM use according to 2019 AGS Beers criteria (*N* = 320).

Variables	Overall	Multimorbidity (*n* = 189, 59.1%)	Polypharmacy (*n* = 77, 24.1%)	PIM ^†^ Use (*n* = 151, 47.2%)
Age (years)	71.9 (SD: 6.07)	71.6 (SD: 5.98)	71.5 (SD: 6.24)	72.8 (SD: 6.14)
65–74	209 (65.3)	124 (38.8)	49 (15.3)	93 (29.1)
≥75	111 (34.7)	65 (20.3)	28 (8.8)	58 (18.1)
Men	189 (59)	106 (33.1)	48 (15)	93 (29)
Women	131 (41)	83 (25.9)	29 (9)	58 (18.1)
Married	225 (70.3)	128 (40)	58 (18.1)	104 (32.5)
Illiterate	208 (65)	112 (35)	25 (7.8)	51 (15.9)
Overweight/Obese	14 (4.4)	9 (2.8)	5 (1.6)	6 (1.9)
Hospitalization previous year	126 (39.4)	80 (25)	35 (10.9)	64 (20)
Serum creatinine (micromol/L) (*n* = 188, 58.7%)	69.6 (SD: 27.6)	68.1 (SD: 28.2)	66.0 (SD: 27.4)	70.8 (SD: 28.8)
Creatine clearance < 30 mL/min	42 (13.1)	30 (9.4)	13 (4.1)	21 (6.6)
**Charlson’s comorbidity index (score)**	2.53 (SD: 1.38)	3.1 (SD: 1.36)	2.54 (SD: 1.61)	2.25 (SD: 1.40)
Mild (1–2 points)	170 (53.1)	57 (17.8)	41 (12.8)	85 (26.6)
Moderate (3–4 points)	136 (42.5)	120 (37.5)	31 (9.7)	59 (18.4)
Severe (≥ 5 points)	14 (4.4)	12 (0.6)	5 (1.6)	7 (2.2)
**Comorbidities**				
Hypertension	213 (66.6)	157 (49.1)	54 (16.9)	100 (31.3)
Diabetes	118 (36.9)	103 (32.2)	27 (8.4)	52 (16.3)
Dyslipidemia	40 (12.5)	38 (11.9)	16 (5.0)	23 (7.2)
Coronary heart disease	31 (9.7)	28 (8.8)	5 (1.6)	16 (5.0)
Peptic ulcer disease	30 (9.4)	28 (8.8)	4 (1.3)	10 (3.1)
Congestive heart failure	11 (3.4)	9 (2.8)	2 (0.6)	5 (1.6)
Pneumonia	10 (3.1)	9 (2.8)	3 (0.9)	6 (1.9)
HIV	6 (1.9)	5 (1.6)	3 (0.9)	3 (0.9)
Other diseases	72 (22.5)	58 (18.1)	18 (5.6)	31 (9.7)
Number of medications	3.4 (SD: 1.69)	3.5 (SD: 1.66)	5.8 (SD: 1.24)	3.4 (SD: 1.72)
Comprehensive geriatric assessment *				
Understand verbal and non-verbal communication	233 (72.8)	145 (45.3)	63 (19.7)	92 (28.8)
Physical fitness	272 (85)	159 (49.7)	64 (20)	120 (37.5)
Using walking assistance devices	118 (36.9)	72 (22.5)	41 (12.8)	72 (22.5)
Lack of interest in activities	42 (13.1)	22 (6.9)	14 (4.4)	25 (7.8)
Persistent anger with self/others	51 (15.9)	30 (9.4)	22 (6.9)	24 (7.5)
Cognitive impairment	56 (17.5)	31 (9.7)	14 (4.4)	35 (10.9)
Had repeated health complaints	70 (21.9)	43 (13.4)	21 (6.6)	34 (10.6)
Experienced fall in the past year	44 (13.8)	21 (6.6)	11 (3.4)	21 (6.6)
Flare-ups of pain	56 (17.5)	36 (11.3)	20 (6.3)	26 (8.1)
Health fluctuation/deterioration	50 (15.6)	26 (8.1)	6 (1.9)	30 (9.4)

SD: standard deviation, ^†^ AGS Beers criteria 2019; HIV: human immunodeficiency virus. * selected area.

**Table 2:** Potentially inappropriate medication use among older outpatients according to 2019 AGS Beers criteria (total PIMs = 200).

Therapeutic Category	Drugs	Number of Patients (%)	Recommendation	Quality of Evidence	Strength of Recommendation
Antiparkinsonian agents	Trihexyphenidyl	1 (0.3)	Avoid	Moderate	Strong
Central alpha-agonists	Methyldopa	5 (1.6)	Avoid	Low	Strong
Antidepressants	Amitriptyline	25 (7.8)	Avoid	High	Strong
Cardiovascular agents	Diuretics	32 (10)	Use with caution	Moderate	Strong
	Aspirin	22 (6.9)	Use with caution in adults ≥ 70 years	Moderate	Strong
	RAS inhibitors or potassium-sparing diuretics	17 (5.3)	Avoid use in those with CrCl < 30 mL/min	Moderate	Strong
	Nifedipine	9 (2.8)	Avoid	High	Strong
	Chlorthalidone	9 (2.8)	Use with caution	Moderate	Strong
	Digoxin	5 (1.6)	Avoid dosages > 0.125 mg/day	Moderate	Strong
Endocrine agents	Insulin, sliding scale	28 (8.8)	Avoid	Moderate	Strong
	Glimepiride	15 (4.7)	Avoid	Moderate	Strong
Anti-infective agents	Ciprofloxacin	3 (0.9)	Avoid using when CrCl < 30 mL/min	Moderate	Strong
Gastrointestinal agents	Omeprazole	6 (1.9)	Avoid scheduled use for >8 weeks unless for high-risk patients	High	Strong
	Pantoprazole	3 (0.9)			
Drug–drug interactions	Trimethoprim-sulfamethoxazole + ACE inhibitors	11 (3.4)	Use with caution in patients on ACEI or ARB and decreasedCrCl	Low	Strong
	Prazosin + Furosemide	9 (2.8)	Avoid in older women	Moderate	Strong

RAS: renin–angiotensin system; ACE: angiotensin-convertase enzyme; ARB: angiotensin-receptor blockers; CrCl: creatinine-clearance.

**Table 3:** Univariate and multivariable logistic regression analysis of determinants of multimorbidity, polypharmacy, and PIM exposure in older adults in Ethiopia.

	Multimorbidity		Polypharmacy		PIM Use	
Ageing Characteristics	*Crude OR*	*Adjusted OR*	*Crude OR*	*Adjusted OR*	*Crude OR*	*Adjusted OR*
Understand verbal and non-verbal communication	1.32 (1.02–1.71) *	1.37 (0.97–1.94)	2.04 (1.04–4.02) *	**2.06** (1.02–4.17) *	0.47 (0.34–0.65) **	**0.46** (0.33–0.65) **
Physical fitness ^†^	0.87 (0.65–1.17)	0.95 (0.65–1.39)	0.85 (0.42–1.72)	0.82 (0.39–1.70)	0.68 (0.47–0.97) *	0.69 (0.47–1.00)
Using walking assistance devices	1.01 (0.80–1.27)	1.15 (0.84–1.58)	2.52 (1.47–4.32) **	**2.41** (1.35–4.28) **	1.85 (1.36–2.51) **	**1.83** (1.32–2.53) **
Lack of interest in activities	0.93 (0.67–1.28)	0.90 (0.60–1.34)	1.71 (0.84–3.47)	1.68 (0.81–3.47)	1.48 (1.02–2.15) *	**1.46** (1.00–2.14) *
Persistent anger with self/others	1.08 (0.81–1.45)	1.03 (0.70–1.50)	2.91 (1.55–5.47) **	**3.33** (1.71–6.47) **	1.18 (0.81–1.70)	1.21 (0.83–1.78)
Cognitive impairment	0.83 (0.46–1.48)	0.98 (0.67–1.43)	1.05 (0.53–2.09)	1.00 (0.49–2.05)	1.69 (1.21–2.37) **	**1.65** (1.15–2.34) **
Had repeated health complaints	1.11 (0.86–1.44)	1.07 (0.76–1.50)	1.45 (0.80–2.63)	1.45 (0.78–2.69)	1.31 (0.95–1.80)	1.32 (0.95–1.84)
Experienced fall in the past year	0.71 (0.51–0.99) *	0.71 (0.46–1.08)	1.10 (0.52–2.31)	1.10 (0.51–2.35)	1.01 (0.67–1.51)	0.98 (0.65–1.48)
Flare-ups of pain	1.54 (1.16–2.04) **	**1.64** (1.13–2.39) **	1.94 (1.03–3.67) *	**2.00** (1.03–3.89) *	0.97 (0.66–1.42)	0.96 (0.65–1.43)
Health fluctuation/deterioration	0.89 (0.66–1.21)	0.79 (0.53–1.17)	0.37 (0.15–0.92) *	0.40 (0.16–1.01)	1.58 (1.11–2.24) *	**1.61** (1.11–2.32) **

* *p* < 0.05; ** *p* < 0.01; OR: odds ratio, adjusted for: age, male gender, marital status—married, illiterate, and Charlson comorbidity index; ^†^ ability to perform daily activities normally; blue color: protective factor.

## Data Availability

Data is contained within the article.
